# Microscopic, stereological, and biochemical investigation of the effects of vitamin B12 on vascular changes in an experimental subarachnoid hemorrhage model in rats

**DOI:** 10.1007/s10143-025-03576-0

**Published:** 2025-05-15

**Authors:** Murat Yücel, Eyüp Çetin, Cumaali Demirtaş, Cansu Sönmez, Hakan Beyaztaş, Eray Metin Güler

**Affiliations:** 1 Neurosurgery Clinic, Yalova University Faculty of Medicine, Yalova, Turkey; 2 Department of Neurosurgery, Health Sciences University, Haydarpaşa Numune Health Application and Research Center, Üsküdar, Istanbul, Turkey; 3 Veterinary Clinic, Health Sciences University Hamidiye Experimental Animals Laboratory, Istanbul, Turkey; 4 Pathology Clinic, Health Sciences University Haydarpaşa Numune Hospital, Istanbul, Turkey; 5 Department of Medical Biochemistry, Hamidiye Faculty of Medicine, University of Health Sciences Turkey, Istanbul, Turkey; 6 Department of Medical Biochemistry, Hamidiye Institute of Health Sciences, University of Health Sciences Turkey, Istanbul, Turkey; 7 Department of Medical Biochemistry, Haydarpaşa Numune Health Application and Research Center, University of Health Sciences Turkey, Istanbul, Turkey

**Keywords:** Subarachnoid hemorrhage, Cerebral vasospasm, Vitamin B12

## Abstract

Spontaneous subarachnoid hemorrhage (SAH) is a severe neurological condition with significant morbidity and mortality. Cerebral vasospasm, a common complication of SAH, is a leading cause of delayed ischemic injury. Oxidative stress and inflammation are central to the pathophysiology of vasospasm. Vitamin B12, a water-soluble vitamin with antioxidant and anti-inflammatory properties, has shown potential neuroprotective effects in experimental models. This study aimed to investigate the effects of vitamin B12 on vascular changes, oxidative stress, and inflammatory markers in an experimental rat model of SAH. Eighteen Sprague Dawley rats were divided into three groups: a control group, an SAH group, and an SAH + B12 group. SAH was induced by injecting autologous blood into the cisterna magna. The SAH + B12 group received intraperitoneal B12 (15 mcg/kg) at 1 and 24 h post-SAH. Biochemical parameters, including total oxidant status (TOS), total antioxidant status (TAS), oxidative stress index (OSI), and inflammatory cytokines (IL-1β, IL-6, TNF-α), were analyzed. Histopathological evaluation of the basilar artery was performed, measuring arterial diameter and wall thickness. The SAH group exhibited significant elevations in TOS, OSI, IL-1β, IL-6, and TNF-α levels, along with decreased TAS, indicating heightened oxidative stress and inflammation. The SAH + B12 group showed significant reductions in TOS, OSI, and inflammatory cytokines compared to the SAH group (*p* < 0.05), alongside improved TAS levels. Histopathological findings demonstrated reduced arterial wall thickening and preserved lumen diameter in the SAH + B12 group compared to the untreated SAH group. Although the differences in arterial diameter and wall thickness were not statistically significant, the findings suggest that B12 may mitigate SAH-induced vascular injury by reducing oxidative stress and inflammation. These results highlight B12's potential as a therapeutic agent for SAH-related vasospasm. Further studies are needed to validate these findings in larger populations and clinical settings.

## Introduction

Spontaneous subarachnoid hemorrhage (SAH) is a serious neurological condition with an annual incidence of 6–11 per 100,000 individuals. This condition is associated with significant mortality and morbidity, as approximately 50% of cases are fatal [1, 2]. Most SAH cases result from the rupture of an intracranial aneurysm, accounting for 85%. This rupture leads to bleeding into the subarachnoid space, causing elevated intracranial pressure and potential ischemic damage to brain tissue. The prognosis for SAH remains poor, causing significant challenges for patients and healthcare systems [2, 3]. SAH is most commonly observed in individuals aged 60 years and older, with this age group being particularly vulnerable to its severe outcomes. In terms of stroke epidemiology, SAH accounts for 5% of all cases, highlighting its clinical importance [4]. Mortality from SAH is approximately 30%, and of those who survive, 40–50% experience long-term morbidity. Only 20% of patients fully recover and regain their pre-hemorrhage functional status, which underscores the devastating nature of this condition [5].

Cerebral vasospasm, a common SAH complication, involves transient narrowing of cerebral arteries. This narrowing can reduce cerebral perfusion, leading to delayed ischemic neurological deficits. Vasospasm typically occurs 3–7 days after hemorrhage and resolves within two weeks [6]. Angiographic vasospasm is observed in 70–80% of SAH patients, while clinical vasospasm, characterized by symptomatic ischemia, occurs in about 30% of cases [7]. The occurrence of cerebral infarction in SAH patients due to vasospasm has been reported in 26% of cases, making it a leading cause of mortality and long-term disability [8]. Despite significant research over the past decades, the exact mechanisms underlying vasospasm remain incompletely understood, complicating the development of effective treatments [9].

Numerous studies have revealed that vasospasm has a multifactorial pathogenesis involving both inflammatory and oxidative pathways. Inflammatory cytokines such as tumor necrosis factor-alpha (TNF-α), interleukin 1-beta (IL-1β), and interleukin 6 (IL-6) play critical roles in initiating and perpetuating inflammation through the MyD88-nuclear factor kB (NF-kB) signaling pathway. This cascade leads to vascular inflammation and endothelial dysfunction, contributing to the development of vasospasm [10, 11]. Additionally, oxidative stress is a key factor in the pathophysiology of vasospasm. The oxidation of oxyhemoglobin in the subarachnoid space generates reactive oxygen species such as superoxide anion radicals and hydroxyl radicals, which trigger lipid peroxidation and cause significant damage to vascular walls. This oxidative damage further exacerbates vasospasm and its clinical consequences [12, 13]. The interplay of these pathways not only highlights the complexity of vasospasm but also underscores the need for targeted therapeutic approaches to address these mechanisms.

### Vitamin B12

Vitamin B12, also known as cobalamin, is a water-soluble vitamin that serves as a cofactor for a limited but critical number of enzymatic reactions. Its unique structure allows it to function in various metabolic processes, including DNA synthesis, red blood cell production, and the maintenance of neurological function [14]. Unlike most water-soluble vitamins, B12 cannot be synthesized by plants; it is primarily obtained from animal-based foods or synthesized de novo by certain bacteria, making dietary intake and proper absorption essential for maintaining adequate B12 levels in the body [15]. The effects of B12 deficiency are most prominent in the hematopoietic and nervous systems. In the central nervous system (CNS), B12 plays a crucial role in neuronal and glial cell functions. Astrocytes, the most abundant glial cells in the CNS, rely on B12 to regulate cytokine production, neurotransmitter uptake, ion transport, and the integrity of the blood–brain barrier [16]. Deficiency disrupts these critical processes, leading to neuronal injury, neuroinflammation, and impaired synaptic transmission, with chronic deficiency potentially causing structural damage to white matter, leading to long-term cognitive and motor deficits [17, 18].

B12 deficiency is closely linked to elevated homocysteine levels, a significant risk factor for ischemic cerebrovascular diseases. Homocysteine, a sulfur-containing amino acid produced during methionine metabolism, accelerates atherosclerosis and contributes to vascular diseases, including stroke. Elevated homocysteine levels can result from genetic enzyme defects or deficiencies in cofactors such as folic acid, B6, and B12, which are essential for homocysteine metabolism. These elevated levels promote oxidative stress and vascular inflammation, exacerbating the risk of thrombotic and ischemic events [19, 20]. Studies have shown that therapeutic combinations of folic acid, B6, and B12 effectively reduce plasma homocysteine levels. However, further research is needed to assess their efficacy in reducing vascular diseases [19].

B12 has demonstrated neuroprotective and antioxidant properties, reducing oxidative stress, inhibiting pro-inflammatory cytokines, and enhancing neuronal repair. By acting on multiple pathways involved in vascular and neuronal injury, B12 has shown potential as a therapeutic agent for various neurological conditions, including those involving vascular compromise [20]. Its role in mitigating oxidative damage and inflammation positions it as a promising candidate for addressing the multifactorial pathogenesis of vasospasm following SAH.

Despite extensive research on SAH and its complications, the role of B12 in this context remains largely unexplored. Existing studies on vasospasm have primarily focused on pharmacological agents targeting single pathways, such as calcium channel blockers or anti-inflammatory drugs. However, these interventions have shown limited efficacy in addressing the complex interplay of inflammatory and oxidative pathways involved in vasospasm. Given the multifactorial nature of vasospasm, B12, which can simultaneously target inflammation and oxidative stress, holds significant potential.

We hypothesize that B12 can mitigate cerebral vasospasm by reducing the production of spasmogenic cytokines and free radicals, thereby preserving vascular integrity and cerebral perfusion. This study aims to investigate the effects of B12 on vascular changes in an experimental model of subarachnoid hemorrhage in rats. Through a combination of biochemical, histological, and stereological analyses, this research seeks to elucidate the mechanisms by which B12 exerts its protective effects. By addressing the gaps in current knowledge, this study has the potential to pave the way for novel therapeutic strategies in the management of SAH and its complications [20].

## Materials and methods

Sprague Dawley female rats weighing an average of 200–250 g were used. The study included three groups, each consisting of six rats. The first group served as the control group with no SAH induced, and the aim was to demonstrate the normal anatomy of the basilar artery and establish baseline levels for biochemical and histopathological parameters. The second group included rats in which SAH was induced but no B12 was administered. The third group included rats in which SAH was induced and intraperitoneal B12 (15 mcg/kg) was administered at 1 h and 24 h post-SAH. All animals were sacrificed at the same time point—48 h after SAH induction—to ensure consistency in biochemical and histological comparisons across groups.

All animals were randomly assigned to the experimental groups using a computer-generated randomization protocol prior to any intervention. Additionally, all investigators involved in outcome assessment, including biochemical and histopathological analyses, were blinded to group allocation throughout the study to reduce bias and ensure objective data interpretation.

For SAH induction, the rats were fasted for 12 h before undergoing general anesthesia. Ketamine Hydrochloride (Ketalar, Parke-Davis, Eczacıbaşı İlaç Sanayi, İstanbul) was administered intraperitoneally at a dose of 50 mg/kg, along with Xylazine (Rhompun Injectable, Bayer Türk Kimya Sanayi, İstanbul) at a dose of 10 mg/kg. After the experiments, the rats were allowed to recover in room air, maintained at a temperature of 20–22 °C, and fed with standard rat feed and adequate water.

SAH was induced using a percutaneous 30G needle inserted into the cisterna magna to withdraw 0.2 ml of cerebrospinal fluid (CSF), which was then replaced with 0.2 ml of autologous blood obtained from the femoral artery. A single hemorrhage model was utilized. The presence of vasospasm in the SAH-induced rats was clinically evaluated until the time of sacrifice using the Bederson scoring system and Garcia scoring system.

For the sacrifice procedure, the rats were euthanized under general anesthesia by cervical dislocation, followed by intracardiac blood collection. The sacrificed rats underwent frontoparieto-occipital craniectomy to remove intracranial structures from the foramen magnum without disrupting anatomical integrity. These structures were fixed in 10% formaldehyde solution for 24 h. The brainstem and basilar artery were then subjected to tissue processing for 16 h, embedded in paraffin blocks, and sectioned into 5-µm slices. The sections were stained with hematoxylin and eosin (H&E). The slides were examined under a light microscope by two independent observers blinded to the groups, and the basilar artery volume and lumen volume were measured. The recorded results were statistically analyzed using the One-Way ANOVA method.

### Histopathological examination

At the end of the study, the rats were anesthetized with a ketamine-xylazine combination. Once the loss of the tail pinch reflex was confirmed, the rats were euthanized using the decapitation method. Following sacrifice, the rats underwent frontoparieto-occipital craniectomy, and intracranial structures up to the foramen magnum were removed without disrupting their anatomical integrity. These tissues were fixed in 10% buffered formaldehyde solution. Subsequently, they were subjected to routine tissue processing procedures and embedded in paraffin. From the paraffin blocks, 5-µm-thick sections were prepared and stained with H&E. The stained sections were examined under a light microscope by two independent observers who were blinded to the groups. Measurements of the basilar artery lumen area and wall thickness were recorded. The results were analyzed statistically using the One-Way ANOVA method.

### Biochemical parameters examination

Biochemical parameters were analyzed in serum and in the supernatant obtained from homogenized tissues. All parameters measured in the tissues were normalized according to their protein concentrations.

### Collection of blood samples

At the end of the experimental procedure, approximately 3 mL of intracardiac blood was collected from the animals into biochemistry tubes with clot activators. The samples were centrifuged at 3000 × g for 10 min, and the serum was separated. Serum samples were stored at ‒80 °C until biochemical analyses were performed.

### Tissue homogenization

For biochemical analyses, approximately 1 mg of frontal tissue was weighed and placed into dry tubes. A 1:10 ratio of 1 × PBS was added to the samples, which were then homogenized using ceramic beads. The homogenate was centrifuged at + 4 °C at 10,000 × g for 30 min, and the supernatant was transferred to a separate tube. The total protein concentration of the supernatant obtained from tissue homogenization was determined using the Bradford method.

### Oxidative stress biomarkers

TAS and TOS levels of the samples were measured using commercially available kits with a photometric method. The OSI was calculated as the ratio of TOS to TAS. The TAS and TOS levels were measured using Rel Assay Diagnostics kits (Gaziantep, Turkey), following the manufacturer’s instructions. The assays were performed using a spectrophotometric method, and results were expressed as mmol Trolox equivalent/L (for TAS) and μmol H₂O₂ equivalent/L (for TOS). The OSI was calculated using the formula: OSI = (TOS/TAS) × 10. Although superoxide dismutase (SOD) and glutathione peroxidase (GPx) activities were not measured individually, their contributions to the overall antioxidant capacity are reflected within the TAS measurement.

### Enzyme-linked immunosorbent assay (ELISA)

Serum and tissue levels of IL-1β, IL-6, and TNF-α were quantified using commercially available ELISA kits (Bioassay Technology Laboratory, Shanghai, China). The assays were performed photometrically at 450 nm, and concentrations were calculated using standard calibration curves provided by the manufacturer. Results are reported in pg/mL. The levels of IL-1β, IL-6, TNF-α, M30, and M65 in the serum and tissues were measured using commercially purchased enzyme-linked immunosorbent assay (ELISA) kits via a photometric method. The measured levels of IL-1β, IL-6, TNF-α, M30, and M65 were determined by comparing the results with pre-established standard curves.

### Ethical compliance

All experimental procedures complied with Directive 2010/63/EU of the European Parliament on the protection of animals used for scientific purposes. The study protocol was reviewed and approved by the Hamidiye University Ethics Committee.

## Results

### Statistical method

Descriptive statistics for the data included mean, standard deviation, median, minimum, maximum, frequency, and ratio values. The distribution of variables was assessed using the Kolmogorov–Smirnov and Shapiro–Wilk tests. For the analysis of quantitative independent variables that did not follow a normal distribution, the Kruskal–Wallis and Mann–Whitney U tests were employed. All analyses were performed using the SPSS 27.0 software. All cytokine levels (IL-1β, IL-6, TNF-α) are expressed in pg/mL. TAS is expressed in mmol Trolox equivalent per liter (mmol Trolox equiv./L), TOS in micromoles of hydrogen peroxide equivalent per liter (μmol H₂O₂ equiv./L), and OSI as an arbitrary unit calculated from the TOS/TAS ratio.

The diameter and wall thickness of the basilar artery provide crucial insights into the extent of vascular damage and recovery following SAH. In the control group, the basilar artery maintained a normal diameter and wall thickness, serving as a reference for healthy vasculature. The SAH group exhibited significant narrowing of the artery diameter and increased wall thickness, indicative of vasospasm, a hallmark pathological response to SAH (*p* < 0.001 compared to the control group). Although the SAH + B12 group showed a trend toward increased luminal diameter and reduced wall thickness compared to the SAH group, these differences did not reach statistical significance (*p* > 0.05). These observations suggest a potential, though not statistically confirmed, effect of B12 in mitigating vasospasm-related changes, possibly via its anti-inflammatory and antioxidant mechanisms (Table 1).

**Table 1:** Descriptive statistics of oxidative stress markers: inflammatory cytokines, and vascular parameters in brain tissue and serum samples

		Min-Max			Median	Mean ± SD/n-%		
Brain Tissue								
Basilar Artery Diameter (µm)		89.3	-	226.8	136.1	140.5	±	34.2
Arterial Wall Thickness (µm)		24.5	-	62.1	35.1	38.0	±	10.5
Total Oxidant Status (TOS)		2.8	-	9.8	6.4	5.6	±	2.1
Total Antioxidant Status (TAS)		0.2	-	0.8	0.6	0.6	±	0.2
Oxidative Stress Index (OSI)		3.7	-	38.1	11.4	12.1	±	9.5
Interleukin-1 Beta (IL-1β)		4.4	-	10.9	8.5	8.3	±	1.9
Interleukin-6 (IL-6)		1.6	-	5.2	3.9	3.5	±	1.3
Tumor Necrosis Factor Alpha (TNF-α)		37.3	-	142.4	105.1	96.0	±	33.9
Hypoxia-Inducible Factor 1 Alpha (HIF-1α)		0.3	-	1.2	0.8	0.8	±	0.3
Cytokeratin 18-M65 (CK18-M65)		110.3	-	490.9	309.1	277.0	±	104.0
***Serum***								
Total Oxidant Status (TOS)		3.9	-	10.5	8.6	7.8	±	2.0
Total Antioxidant Status (TAS)		0.3	-	1.2	0.6	0.7	±	0.3
Oxidative Stress Index (OSI)		3.5	-	27.2	16.1	14.2	±	7.2
Interleukin-1 Beta (IL-1β)		7.3	-	27.7	21.2	18.5	±	7.4
Interleukin-6 (IL-6)		5.4	-	29.9	22.7	19.9	±	8.2
Tumor Necrosis Factor Alpha (TNF-α)		91.8	-	409.8	289.5	258.4	±	104.8
Hypoxia-Inducible Factor 1 Alpha (HIF-1α)		1.6	-	5.8	3.7	3.6	±	1.5
Cytokeratin 18-M65 (CK18-M65)		88.1	-	444.9	316.1	282.4	±	120.9
Total Thiol (µmol/L)		223.8	-	569.1	349.5	364.4	±	97.0
Native Thiol (µmol/L)		70.2	-	393.4	123.3	185.4	±	127.2
Disulfide (µmol/L)		41.5	-	141.7	89.8	89.5	±	28.1
% Native Thiol/Total Thiol		21.7	-	80.9	36.5	46.6	±	21.3
% Disulfide/Total Thiol		9.5	-	39.1	31.7	26.7	±	10.6
% Disulfide/Native Thiol		11.8	-	180.2	87.0	79.0	±	53.2
Group	Control					6		33.3%
	SAH Group					6		33.3%
	SAH + B12 Group					6		33.3%

Oxidative stress plays a pivotal role in the pathogenesis of SAH-related complications. In the SAH group, elevated TOS and OSI highlighted the imbalance between oxidant production and antioxidant defense mechanisms (*p* < 0.001 compared to the control group). Concurrently, a decrease in TAS underscored the depletion of antioxidant reserves in response to heightened oxidative stress. Treatment with B12 in the SAH + B12 group significantly reduced TOS (*p* < 0.05) and OSI levels while improving TAS (*p* < 0.05) compared to the SAH group, reinforcing its role as a potent antioxidant capable of neutralizing reactive oxygen species and restoring redox homeostasis (Table 1).

Inflammatory cytokines such as IL-1β, IL-6, and TNF-α were markedly elevated in the SAH group, reflecting an acute inflammatory response to vascular injury (*p* < 0.001 compared to the control group). These cytokines exacerbate endothelial damage, promote vasospasm, and worsen neurological outcomes. IL-6 levels were significantly reduced in the SAH + B12 group, while IL-1β and TNF-α showed no significant changes. Therefore, B12 may exert partial modulatory effects on the inflammatory cascade (Table 1).

Hypoxia-Inducible Factor 1 Alpha (HIF-1α) is a marker of hypoxic stress in tissues. Elevated HIF-1α levels in the SAH group indicated a hypoxic microenvironment resulting from impaired cerebral blood flow (*p* < 0.001 compared to the control group). In the SAH + B12 group, a reduction in HIF-1α levels was observed (*p* < 0.05) compared to the SAH group, suggesting improved oxygen delivery and reduced hypoxic stress, likely due to the protective effects of B12 on vascular function (Table 1).

Thiol/disulfide homeostasis serves as an indicator of oxidative protein modifications. The SAH group exhibited decreased levels of Total Thiol and Native Thiol alongside increased Disulfide levels and ratios (e.g., % Disulfide/Total Thiol), reflecting oxidative thiol modifications and protein damage (*p* < 0.001 compared to the control group). In the SAH + B12 group, the restoration of thiol/disulfide balance was evident through elevated thiol levels and reduced disulfide formation (*p* < 0.05), highlighting B12’s ability to prevent oxidative damage and maintain protein integrity (Table 1).

Cytokeratin 18-M65 (CK18-M65) is a well-established biomarker of apoptosis and necrosis, with elevated levels indicating extensive cellular damage. In our study, CK18-M65 levels were significantly elevated in the SAH group compared to the control group (*p* < 0.001), reflecting widespread neurovascular injury. Although a reduction in CK18-M65 levels was observed in the SAH + B12 group (*p* = 0.04 vs. SAH), the values remained elevated relative to the control group (*p* < 0.05). This suggests a partial cytoprotective effect of B12, likely mediated by its antioxidant and anti-inflammatory properties (Table 1).

The groups in the study were balanced in terms of sample size, with each group comprising six animals (33.3% of the total sample). The control group served as the baseline, exhibiting normal values for all measured parameters. As expected, the SAH group displayed significant deviations across oxidative stress markers, inflammatory cytokines, and vascular measurements (p < 0.001 compared to the control group), consistent with the known pathophysiology of SAH. The SAH + B12 group demonstrated statistically significant improvements in several biochemical markers, including reductions in OSI and IL-6 (p < 0.05 vs. SAH), along with non-significant trends toward improvement in arterial wall thickness, IL-1β, TNF-α, and CK18-M65 levels. These findings suggest that B12 may have a beneficial, though partially confirmed, effect in attenuating SAH-induced vascular and inflammatory damage (Table 1).

The basilar artery diameter and arterial wall thickness did not show a significant difference (*p* > 0.05) between the Control, SAH, and SAH + B12 groups. The total oxidant status value in the SAH and SAH + B12 groups was significantly higher (*p* < 0.05) compared to the Control group. However, there was no significant difference (*p* > 0.05) in total oxidant status between the SAH and SAH + B12 groups. The TAS value in the Control group was significantly higher (*p* < 0.05) than in the SAH and SAH + B12 groups. There was no significant difference (*p* > 0.05) in TAS between the SAH and SAH + B12 groups. (Table 2).

**Table 2:** Comparison of brain tissue oxidative stress markers: inflammatory cytokines, and vascular parameters among control, SAH and SAH + B12 groups

Brain Tissue		^1^Control Group (*n* = 6)			^2^SAH Group (*n* = 6)			^3^SAH + B12 Group (*n* = 6)			*p*	
**Basilar Artery Diameter (mm)**	**Mean ± SD**	153.0	±	47.5	127.8	±	34.6	140.7	±	12.3	0.476	^K^
	**Median**	147.0			132.0			137.8				
**Arterial Wall Thickness (mm)**	**Mean ± SD**	40.1	±	13.2	40.5	±	12.1	33.5	±	4.6	0.519	^K^
	**Median**	35.1			36.4			33.1				
**Total Oxidant Status (TOS)**	**Mean ± SD**	3.0	±	0.1	7.6	±	1.4	6.3	±	0.6	**0.002**	^K^
	**Median**	3.0^23^			7.0			6.6				
**Total Antioxidant Status (TAS)**	**Mean ± SD**	0.77	±	0.05	0.43	±	0.18	0.57	±	0.06	**0.002**	^K^
	**Median**	0.78			0.47^1^			0.658^1^				
**Oxidative Stress Index (OSI)**	**Mean ± SD**	3.9	±	0.2	21.2	±	11.1	11.3	±	2.1	**0.001**	^K^
	**Median**	3.9^23^			15.1			11.4^2^				
**Interleukin-1 Beta (pg/mL)**	**Mean ± SD**	6.0	±	1.0	10.0	±	0.8	8.8	±	1.0	**0.001**	^K^
	**Median**	6.3^23^			10.1			8.8				
**Interleukin-6 (pg/mL)**	**Mean ± SD**	1.8	±	0.2	4.8	±	0.3	4.0	±	0.6	**0.001**	^K^
	**Median**	1.8^23^			4.7			3.9^2^				
**Tumor Necrosis Factor Alpha (pg/mL)**	**Mean ± SD**	52.8	±	10.8	125.8	±	11.8	109.5	±	11.9	**0.001**	^K^
	**Median**	54.1^23^			125.8			105.1				
**Hypoxia-Inducible Factor 1 Alpha (ng/mL)**	**Mean ± SD**	0.46	±	0.10	0.95	±	0.09	0.90	±	0.17	**0.003**	^K^
	**Median**	0.47^23^			0.95			0.90				
**Cytokeratin 18-M65 (U/L)**	**Mean ± SD**	152.7	±	37.3	357.1	±	78.5	321.1	±	25.3	**0.002**	^K^
	**Median**	143.1^23^			351.3			312.9				

^**K**^Kruskal-Wallis (Mann–Whitney U test)

^1^Significant difference compared to the Control Group (***p* < 0.05**)

^2^Significant difference compared to the SAH Group (***p* < 0.05**)

^3^Significant difference compared to the SAH + B12 Group (***p* < 0.05**)

The OSI value in the SAH and SAH + B12 groups was significantly higher (p < 0.05) compared to the Control group. Additionally, the OSI value in the SAH group was significantly higher (p < 0.05) than in the SAH + B12 group. (Table 2).

The IL-1β value in the SAH and SAH + B12 groups was significantly higher (*p* < 0.05) compared to the Control group. However, there was no significant difference (*p* > 0.05) in IL-1β between the SAH and SAH + B12 groups. The IL-6 value in the SAH and SAH + B12 groups was significantly higher (*p* < 0.05) compared to the Control group. Furthermore, the IL-6 value in the SAH group was significantly higher (*p* < 0.05) than in the SAH + B12 group. (Table 2).

The TNF-α value in the SAH and SAH + B12 groups was significantly higher (*p* < 0.05) compared to the Control group. However, there was no significant difference (*p* > 0.05) in TNF-α between the SAH and SAH + B12 groups. The HIF-1α value in the SAH and SAH + B12 groups was significantly higher (*p* < 0.05) compared to the Control group. However, there was no significant difference (*p* > 0.05) in HIF-1α between the SAH and SAH + B12 groups. The CK 18-M65 value in the SAH and SAH + B12 groups was significantly higher (*p* < 0.05) compared to the Control group. However, there was no significant difference (*p* > 0.05) in CK 18-M65 between the SAH and SAH + B12 groups. (Table 2).

In the SAH and SAH + B12 groups, the total oxidant status value was significantly higher (*p* < 0.05) compared to the control group. The TOS value in the SAH group was also significantly higher (*p* < 0.05) than in the SAH + B12 group (Table 3).

**Table 3:** Comparison of serum oxidative stress markers: inflammatory cytokines, and thiol/disulfide homeostasis among control, SAH and SAH + B12 groups

Serum		^1^Control Group (*n* = 6)			^2^SAH Group (*n* = 6)			^3^SAH + B12 Group (*n* = 6)			*p*	
Total Oxidant Status	Mean ± SD	5.7	±	1.8	9.6	±	0.5	8.1	±	0.9	0.002	^K^
	Median	5.5^23^			9.4			8.3^2^				
Total Antioxidant Status	Mean ± SD	1.03	±	0.17	0.46	±	0.07	0.55	±	0.07	0.002	^K^
	Median	1.10			0.45^1^			0.53^1^				
Oxidative Stress Index	Mean ± SD	5.8	±	2.5	21.5	±	3.4	15.2	±	3.1	0.001	^K^
	Median	4.8^23^			21.6			16.1^2^				
Interleukin-1 Beta (pg/mL)	Mean ± SD	9.2	±	2.1	25.1	±	2.7	21.2	±	3.5	0.001	^K^
	Median	8.1^23^			25.9			22.4				
Interleukin-6 (pg/mL)	Mean ± SD	10.0	±	5.2	27.7	±	2.4	21.9	±	1.7	0.001	^K^
	Median	8.5^23^			28.6			22.7^2^				
Tumor Necrosis Factor Alpha (pg/mL)	Mean ± SD	125.1	±	28.1	339.1	±	56.2	311.1	±	30.4	0.002	^K^
	Median	123.9^23^			346.4			327.9				
Hypoxia-Inducible Factor 1 Alpha (ng/mL)	Mean ± SD	1.7	±	0.1	5.0	±	0.8	4.0	±	0.6	0.001	^K^
	Median	1.6^23^			5.2			3.9^2^				
Cytokeratin 18-M65 (U/L)	Mean ± SD	128.8	±	21.4	382.9	±	49.4	335.6	±	54.6	0.002	^K^
	Median	135.9^23^			384.1			337.6				
Total Thiol (µmol/L)	Mean ± SD	481.2	±	46.5	284.8	±	49.4	327.4	±	42.4	0.002	^K^
	Median	467.2			276.2^1^			323.9^1^				
Native Thiol (µmol/L)	Mean ± SD	356.8	±	26.8	76.6	±	4.8	122.7	±	7.6	0.001	^K^
	Median	358.6			76.0^13^			123.3^1^				
Disulfide (µmol/L)	Mean ± SD	62.2	±	19.1	104.1	±	24.1	102.3	±	19.9	0.015	^K^
	Median	58.7^23^			100.7			104.9				
% Native Thiol/Total Thiol	Mean ± SD	74.5	±	6.2	27.5	±	4.4	37.9	±	4.9	0.001	^K^
	Median	75.4			27.2^13^			36.5^1^				
% Disulfide/Total Thiol	Mean ± SD	12.8	±	3.1	36.3	±	2.2	31.0	±	2.5	0.001	^K^
	Median	12.3^23^			36.4			31.7^2^				
% Disulfide/Native Thiol	Mean ± SD	17.5	±	5.7	135.9	±	30.0	83.5	±	15.7	0.001	^K^
	Median	16.4^23^			134.4			87.0^2^				

^**K**^Kruskal-Wallis (Mann–Whitney U test)

^1^Significant difference compared to the Control Group (***p* < 0.05**)

^2^Significant difference compared to the SAH Group (***p* < 0.05**)

^3^Significant difference compared to the SAH + B12 Group (***p* < 0.05**)

The TAS value in the control group was significantly higher (*p* < 0.05) than in the SAH and SAH + B12 groups. There was no significant difference (*p* > 0.05) in the TAS value between the SAH and SAH + B12 groups (Table 3).

The OSI value was significantly higher (*p* < 0.05) in the SAH and SAH + B12 groups compared to the control group. The OSI value in the SAH group was also significantly higher (*p* < 0.05) than in the SAH + B12 group (Table 3).

The IL-1β value was significantly higher (*p* < 0.05) in the SAH and SAH + B12 groups compared to the control group. There was no significant difference (*p* > 0.05) in the IL-1β value between the SAH and SAH + B12 groups. The IL-6 value was significantly higher (*p* < 0.05) in the SAH and SAH + B12 groups compared to the control group. The IL-6 value in the SAH group was also significantly higher (*p* < 0.05) than in the SAH + B12 group (Table 3).

The TNF-α value was significantly higher (*p* < 0.05) in the SAH and SAH + B12 groups compared to the control group. There was no significant difference (*p* > 0.05) in the TNF-α value between the SAH and SAH + B12 groups (Table 3).

The HIF-1α value was significantly higher (*p* < 0.05) in the SAH and SAH + B12 groups compared to the control group. The HIF-1α value in the SAH group was also significantly higher (p < 0.05) than in the SAH + B12 group (Table 3).

The CK 18-M65 value was significantly higher (*p* < 0.05) in the SAH and SAH + B12 groups compared to the control group. There was no significant difference (*p* > 0.05) in the CK 18-M65 value between the SAH and SAH + B12 groups (Table 3).

The total thiol value in the control group was significantly higher (*p* < 0.05) than in the SAH and SAH + B12 groups. There was no significant difference (*p* > 0.05) in the total thiol value between the SAH and SAH + B12 groups (Table 3).

The native thiol value in the control group was significantly higher (*p* < 0.05) than in the SAH and SAH + B12 groups. The native thiol value in the SAH + B12 group was also significantly higher (*p* < 0.05) than in the SAH group (Table 3).

The disulfide value was significantly higher (*p* < 0.05) in the SAH and SAH + B12 groups compared to the control group. There was no significant difference (*p* > 0.05) in the disulfide value between the SAH and SAH + B12 groups (Table 3).

The % native thiol/total thiol value in the control group was significantly higher (*p* < 0.05) than in the SAH and SAH + B12 groups. The % native thiol/total thiol value in the SAH + B12 group was also significantly higher (*p* < 0.05) than in the SAH group. The % disulfide/total thiol value was significantly higher (*p* < 0.05) in the SAH and SAH + B12 groups compared to the control group. The % disulfide/total thiol value in the SAH group was also significantly higher (*p* < 0.05) than in the SAH + B12 group. The % disulfide/native thiol value was significantly higher (*p* < 0.05) in the SAH and SAH + B12 groups compared to the control group. The % disulfide/native thiol value in the SAH group was also significantly higher (*p* < 0.05) than in the SAH + B12 group (Table 3).

**Fig. 1:**
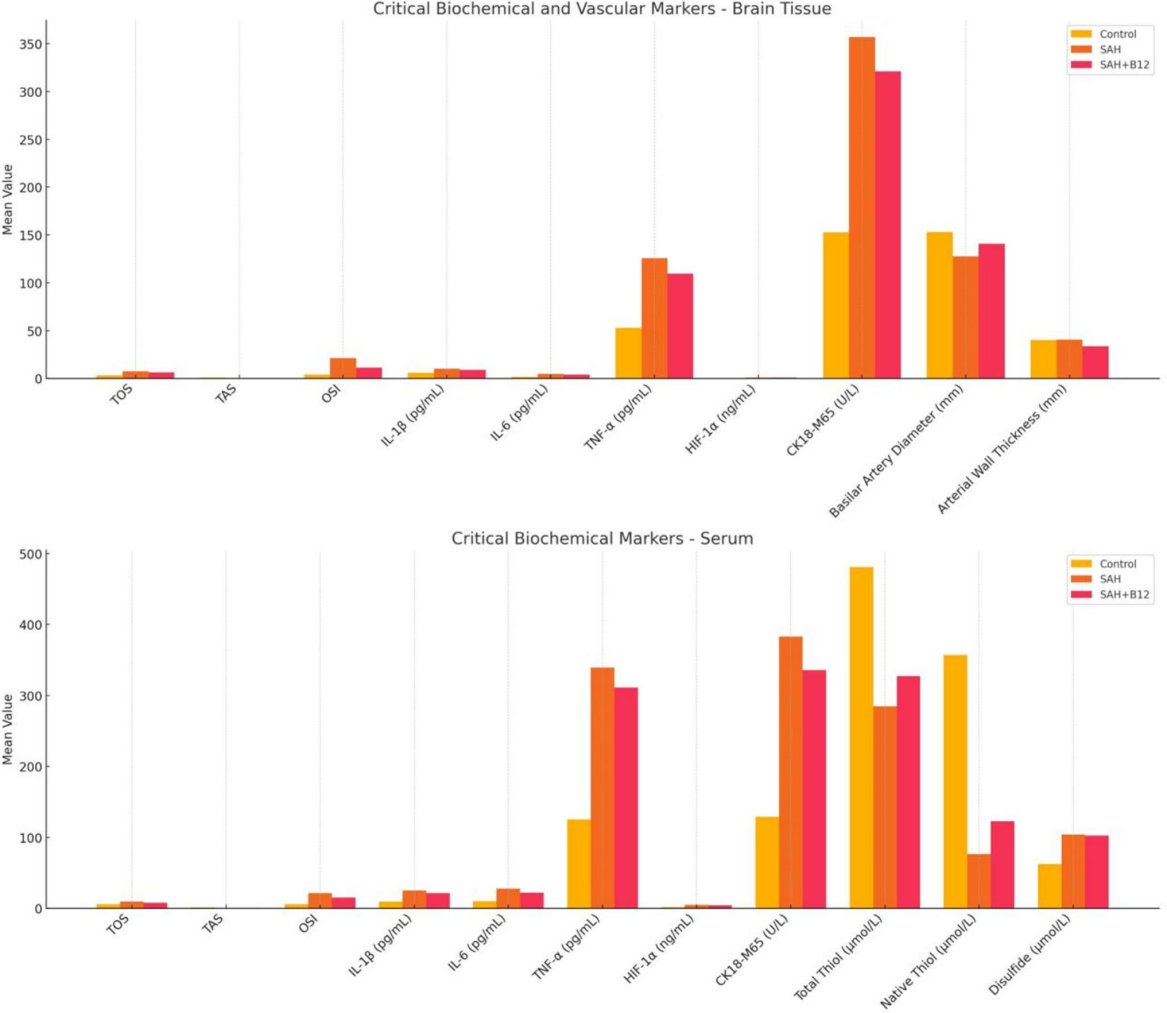
Comparison of biochemical markers across groups

Figure 1 summarizes the biochemical and vascular alterations across Control, SAH, and SAH + B12 groups. In brain tissue, SAH increased oxidative stress and inflammation, with partial reduction following B12 treatment. Serum markers showed similar trends, indicating systemic oxidative and inflammatory responses. Vascular measurements revealed SAH-induced structural changes, which appeared less pronounced in the B12 group.

### Histopathological examination

**Fig. 2:**
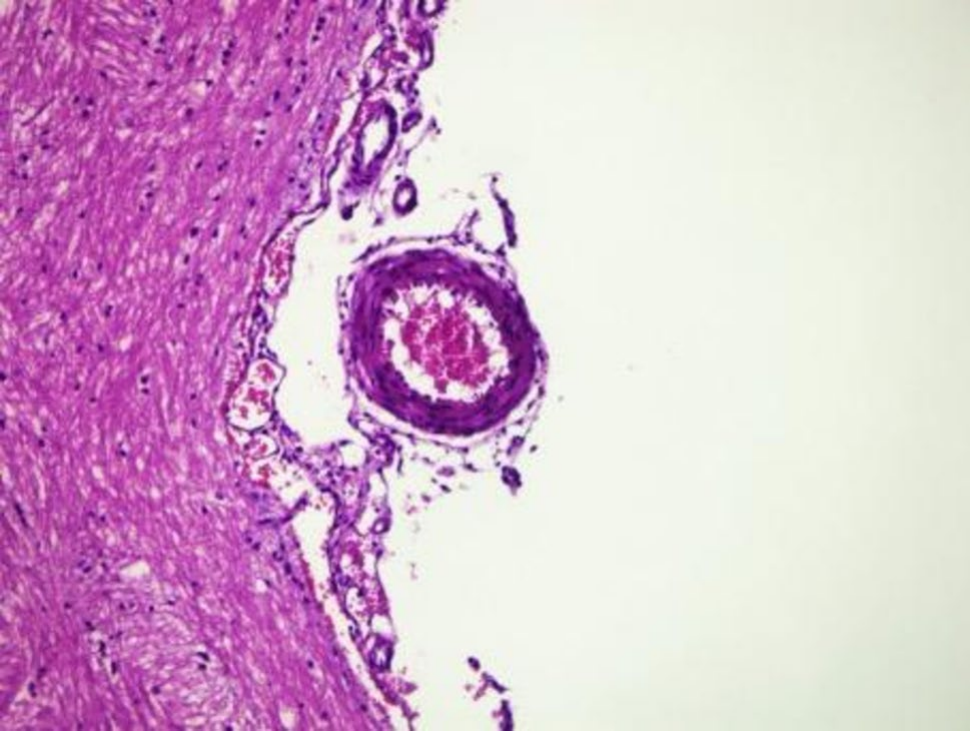
Histopathological examination of the basilar artery in the control group

Histological examination of H&E-stained preparations revealed distinct differences between the control, SAH, and SAH + B12 groups. In the control group (Fig. 2), the basilar artery exhibited a normal histological structure. Light microscopy showed well-defined intima, media, and adventitia layers with no evidence of vascular wall damage or pathological alterations. The surrounding perivascular tissue also maintained its normal architecture, reflecting an absence of inflammation or other histopathological changes. These findings provide a baseline for comparison with the experimental groups.

**Fig. 3:**
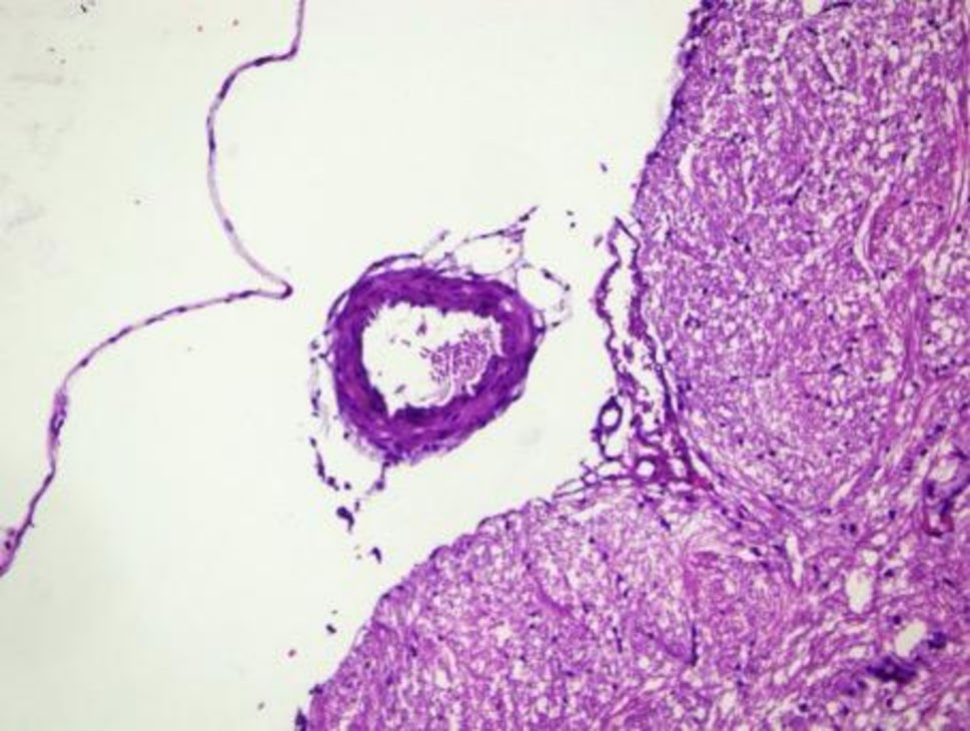
Histopathological evaluation of the basilar artery in the SAH group without treatment

In the SAH group (Fig. 3), significant histopathological changes were observed. Focal undulations in the elastic lamina were noted, along with endothelial cell protrusions extending into the lumen. These changes were indicative of vascular injury and vasospasm, hallmark complications following subarachnoid hemorrhage. The arterial lumen was markedly narrowed, and the vascular wall thickness was increased compared to the control group. These alterations suggest significant structural and functional compromise of the basilar artery, consistent with the expected vascular response to SAH.

**Fig. 4:**
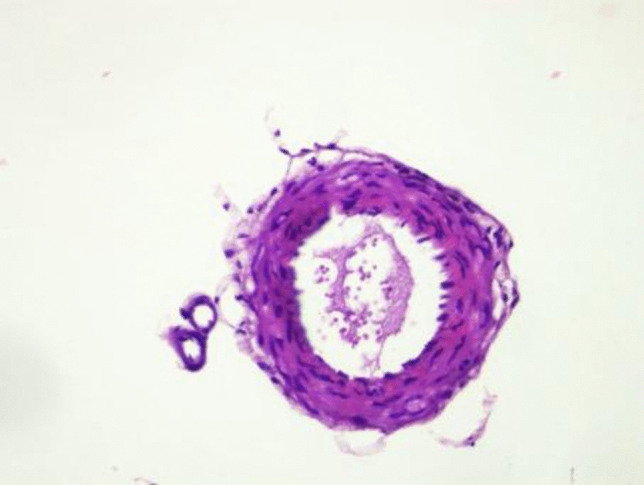
Histopathological assessment of the basilar artery in the SAH + B12 group

In the SAH + B12 group (Fig. 4), the histopathological changes were less severe compared to the untreated SAH group. While some undulations in the elastic lamina and endothelial protrusions were still present, their frequency and severity were notably reduced. The basilar artery lumen appeared more preserved, and vascular wall thickening was less pronounced. These observations suggest that B12 administration may have mitigated the vascular damage and vasospasm associated with SAH. The histological findings in this group align with the hypothesized neuroprotective and anti-inflammatory properties of B12.

The diameters and wall thicknesses of the basilar arteries were quantitatively measured across all groups. These measurements demonstrated significant differences between the groups, highlighting the pathological changes induced by SAH and the potential therapeutic effects of B12. The visual representations provided in Figs. 2, 3, and 4 complement these findings, illustrating the progressive vascular damage from the control group to the SAH group and the relative preservation of vascular architecture in the SAH + B12 group. This progression underscores the importance of early therapeutic intervention in preventing or minimizing SAH-related complications.

## Discussion

Subarachnoid hemorrhage (SAH) is a severe condition characterized by bleeding into the subarachnoid space, often resulting from the rupture of intracranial aneurysms, which account for approximately 85% of spontaneous SAH cases [3, 4]. It has an annual incidence of 6–9 per 100,000 individuals globally, with variations across populations. Finland reports one of the highest rates at 22.5 per 100,000, while China has a lower incidence at 2 per 100,000 [21, 22]. SAH represents 3–5% of all strokes and is associated with significant morbidity and mortality [2, 3]. The condition is influenced by multiple factors, including seasonal, diurnal, and daily patterns, as well as gender and age distribution. While the incidence is higher in younger men, it becomes more common in women after the age of 55 due to hormonal changes and other vascular risk factors [2, 21].

Oxidative stress and inflammation are central to the pathophysiology of SAH. Hemoglobin released into the subarachnoid space following hemorrhage leads to the production of reactive oxygen species (ROS) such as superoxide radicals and hydroxyl radicals [7, 8]. These ROS cause lipid peroxidation and endothelial damage, resulting in vasospasm and delayed cerebral ischemia [6, 7, 9]. Our study showed significantly elevated TOS and OSI in the SAH group, indicating oxidative damage (Table 2) (Fig. 1). B12 supplementation effectively reduced OSI, supporting its role as an antioxidant therapeutic agent [12, 13]. The graphical summary in Fig. 1 condenses these results, providing a clear visual comparison of the oxidative stress markers across the experimental groups.

Inflammatory responses also play a critical role in SAH-induced vasospasm. Elevated levels of pro-inflammatory cytokines such as IL-1β, IL-6, and TNF-α have been widely reported in SAH and are associated with vascular injury and adverse neurological outcomes [23–27]. In Fig. 1, we can clearly see the significant reduction in IL-6 levels in the SAH + B12 group, while IL-1β and TNF-α levels remained elevated. This suggests that B12 may have a partial modulatory effect on the inflammatory cascade (Fig. 1, Table 2 and 3). In our study, these cytokines were significantly elevated in the SAH group compared to controls. B12 administration was associated with a statistically significant reduction in IL-6 levels. However, IL-1β and TNF-α levels remained elevated in the SAH + B12 group and did not differ significantly from the untreated SAH group, suggesting only a limited modulatory effect of B12 on these specific inflammatory mediators (Table 2 and 3, Fig. 4) [28–32]. This partial anti-inflammatory profile highlights the need for further studies to delineate the cytokine-specific effects of B12.

B12, a complex water-soluble vitamin, is essential for neuronal and vascular health. It acts as a cofactor for key enzymatic reactions, including homocysteine metabolism, which is critical for vascular function [10, 11, 15]. Elevated serum homocysteine levels are a recognized risk factor for ischemic cerebrovascular diseases, including stroke [19, 33]. Clarke et al. demonstrated that elevated homocysteine levels are associated with a higher prevalence of peripheral vascular disease (28%), cerebrovascular disease (42%), and coronary artery disease (30%) [34]. B12, combined with folic acid and B6, effectively reduces homocysteine levels [16, 34, 35]. Our study further supports these findings by demonstrating reduced oxidative stress and inflammation in the B12-treated group [12, 14, 26].

Histopathological analysis revealed normal arterial wall structures in the control group, including intact intima, media, and adventitia layers [7, 9]. In contrast, the SAH group exhibited significant vascular wall thickening and luminal narrowing, consistent with vasospasm [3, 27]. These changes were less pronounced in the SAH + B12 group, suggesting a protective role of B12 in ameliorating vascular injury. These findings align with prior studies that reported the neuroprotective and vasodilatory effects of B12 [25, 29].

Astrocytes, the predominant glial cells in the central nervous system, play critical roles in maintaining blood–brain barrier integrity, regulating neuronal metabolism, and modulating inflammatory responses [10, 30]. B12 deficiency disrupts critical processes, causing neuronal injury, neuroinflammation, and impaired synaptic transmission [13, 32]. Chronic deficiency can lead to white matter damage, resulting in long-term cognitive and motor deficits [28, 29].

In conclusion, B12 supplementation reduces oxidative stress, lowers inflammatory cytokines, and improves histopathological changes in SAH. Though the differences in arterial diameter and wall thickness were not statistically significant, the trends suggest that B12 may benefit vasospasm treatment. These findings warrant further investigation in larger-scale studies to fully elucidate the therapeutic potential of B12 in SAH management.

## Conclusion

Although the differences in arterial diameter and wall thickness were not statistically significant, trends suggest that B12 may help treat vasospasm. By mitigating oxidative stress and inflammation, as demonstrated by the significant improvements in oxidative stress markers (such as TOS, TAS, and OSI) and partial reductions in inflammatory cytokines (e.g., IL-6) in Fig. 1, B12 shows promise as a therapeutic agent for SAH-related complications. These findings underscore the potential of B12 in preserving vascular integrity and reducing secondary ischemic injury following SAH. Further large-scale studies are needed to validate these findings and optimize B12 dosing and timing in clinical settings.

This study has several limitations. The small sample size may have reduced the statistical power to detect significant differences in some outcomes, such as arterial diameter and wall thickness. The use of an experimental rat model, while valuable, may not fully replicate the complexity of SAH in humans, warranting translational studies and clinical trials. Additionally, the study did not include neurological function scoring or behavioral assessments, which are essential to evaluate functional recovery in SAH models. Although biochemical and histological parameters provide important mechanistic insights, functional outcomes remain critical to the translational relevance of such interventions. Future research should incorporate comprehensive neurological scoring systems alongside biochemical and histological evaluations to better assess the therapeutic potential of B12.

Additionally, this study focused on biochemical and histopathological changes but did not assess functional neurological outcomes or long-term recovery. Future research should explore these aspects and evaluate dose–response relationships and timing of B12 administration to optimize therapeutic protocols. Finally, further studies are needed to clarify the molecular mechanisms underlying the observed protective effects of B12.

## Data Availability

No datasets were generated or analysed during the current study.
